# Aberrant DNA methylation of miRNAs in Fuchs endothelial corneal dystrophy

**DOI:** 10.1038/s41598-019-52727-z

**Published:** 2019-11-08

**Authors:** Peipei Pan, Daniel J. Weisenberger, Siyu Zheng, Marie Wolf, David G. Hwang, Jennifer R. Rose-Nussbaumer, Ula V. Jurkunas, Matilda F. Chan

**Affiliations:** 1 Department of Ophthalmology, University of California, San Francisco, San Francisco, CA USA; 2 Department of Biochemistry and Molecular Medicine, University of Southern California, Los Angeles, CA USA; 3 Francis I. Proctor Foundation, University of California, San Francisco, CA USA; 4 Department of Ophthalmology, Harvard Medical School, and Schepens Eye Research Institute, Massachusetts Eye and Ear, Boston, MA USA

**Keywords:** Microarray analysis, DNA methylation

## Abstract

Homeostatic maintenance of corneal endothelial cells is essential for maintenance of corneal deturgescence and transparency. In Fuchs endothelial corneal dystrophy (FECD), an accelerated loss and dysfunction of endothelial cells leads to progressively severe visual impairment. An abnormal accumulation of extracellular matrix (ECM) is a distinctive hallmark of the disease, however the molecular pathogenic mechanisms underlying this phenomenon are not fully understood. Here, we investigate genome-wide and sequence-specific DNA methylation changes of miRNA genes in corneal endothelial samples from FECD patients. We discover that miRNA gene promoters are frequent targets of aberrant DNA methylation in FECD. More specifically, *miR-199B* is extensively hypermethylated and its mature transcript miR-199b-5p was previously found to be almost completely silenced in FECD. Furthermore, we find that miR-199b-5p directly and negatively regulates Snai1 and ZEB1, two zinc finger transcription factors that lead to increased ECM deposition in FECD. Taken together, these findings suggest a novel epigenetic regulatory mechanism of matrix protein production by corneal endothelial cells in which *miR-199B* hypermethylation leads to miR-199b-5p downregulation and thereby the increased expression of its target genes, including *Snai1* and *ZEB1*. Our results support miR-199b-5p as a potential therapeutic target to prevent or slow down the progression of FECD disease.

## Introduction

Corneal transparency is critical for good visual acuity. The corneal endothelium regulates the hydration status of the cornea and has an essential role in maintaining corneal deturgescence and preventing edema that can degrade corneal transparency. It is the innermost layer of the cornea and is composed of a single layer of cells that pump excess fluid out of the cornea through active ion-transport processes^1,2^.

Fuchs endothelial corneal dystrophy (FECD) is a bilateral, slowly progressive disorder in which the corneal endothelial cells are diseased and become less efficient at removing fluid. As a result, the highly ordered arrangement of collagen fibers in the corneal stromal layer become disrupted, leading to corneal opacification and vision loss^3^. Other clinical phenotypic changes that occur in FECD include an excessive accumulation of extracellular matrix (ECM), formation of central excrescences (corneal guttae), thickening of Descemet’s membrane, and corneal scarring^4^. At earlier stages of FECD, the formation of corneal guttae can cause light scatter and optical aberrations that can impair vision, even in the absence of overt corneal edema. In later FECD, overt endothelial dysfunction and resultant corneal edema contribute significantly to visual loss. Corneal endothelial cells are largely non-regenerative *in vivo* and their loss is often irreversible^5^. Medical management is often inadequate and corneal endothelial transplantation remains the main therapeutic option to restore vision in patients with advanced FECD. FECD is a leading indication for corneal transplantation in the United States^6^.

FECD is a multi-factorial disease that is associated with a variety of reported spontaneous and inherited mutations^7,8^ and can manifest as both early- and late-onset forms^9^. Mutations in the more common late-onset FECD have been identified in several genes including *SLC4A11*^10,11^, *ZEB1* (also named as *TCF8*)^12^, *LOXHD1*^13^, and *AGBL1*^14^. An expanded trinucleotide repeat in the third intron of transcription factor 4 (*TCF4*, also referred to as E2-2) has also been found to be strongly associated with many cases of late-onset FECD^15,16^. In addition to genetic variations, environmental factors such as oxidative stress have also been implicated in the pathogenesis of FECD through complex cellular and biochemical responses^17–20^. The external location of the cornea renders it directly exposed to the environment and thus more susceptible to external stimuli.

DNA methylation is an epigenetic change that facilitates cellular adaptation to changing environments and has repeatedly been linked to human diseases and aging^21–23^. This has raised interest in understanding the potential contribution of DNA methylation to the development of late-onset eye diseases such as glaucoma^24,25^, age-related macular degeneration^26–28^, and cataract^29^. Epigenetic factors may help explain the phenotypic variation seen amongst cohorts with identical genotypes, including in FECD. We previously identified DNA methylation changes that occur in the corneal endothelial tissue of patients with late-onset FECD using a genome-wide DNA methylation array^30^. Furthermore, many of these changes occurred in microRNA (miRNA) sequences^30^.

Multiple studies over the past decade have demonstrated that miRNAs profoundly influence the cellular responses of tissues to physiologic and pathophysiologic stresses in multiple disease states^31,32^. Stress-dependent regulation can involve upregulation or downregulation of miRNA expression and lead to downstream signaling effects on mRNA targets^31^. Accumulating evidence from human and animal studies have shown that DNA methylation-associated silencing of miRNAs contributes to disease pathogenesis^33^. In the present study, we further analyzed the subset of miRNA data to test the hypothesis that aberrant DNA methylation of miRNAs contributes to FECD pathogenesis. We found that the majority of differentially methylated miRNAs display promoter DNA hypermethylation in FECD. Moreover, we identified *miR-199B* to be extensively hypermethylated in FECD. Using *in silico* and functional assays, we determined that miR-199b-5p directly targets and negatively regulates the expression of two key transcription factors that control ECM production in FECD. Taken together, these findings demonstrate a novel mechanism of epigenetic regulation of ECM production in FECD pathogenesis and identifies miR-199b-5p as a potential clinical biomarker of disease.

## Results

### Global DNA methylation patterns of miRNA sequences are altered in FECD

Our prior genome-scale analysis of the DNA methylation landscape of corneal endothelial tissue found a significant difference between FECD and normal control patients^30^. In particular, we identified a high number of differentially methylated miRNA sequences^30^. Because DNA methylation plays a central role in regulating miRNA expression^34,35^ and widespread miRNA downregulation has been observed in FECD^36^, we performed a subanalysis of the Illumina Infinium HumanMethylation450 (HM450) array data to focus on the 2,227 miRNA probes (targeting 463 miRNA genes in total). Sample pairwise correlation and hierarchical clustering analyses revealed differential genome-wide miRNA DNA methylation patterns in FECD samples compared with normal control samples (Supplementary Fig. 1). Further nonparametric principle component analyses showed that this variance was not attributable to the clinical variables of age, sex, pachymetry, or guttata grading (data not shown).

### The majority of differentially methylated miRNAs display promoter DNA hypermethylation in FECD

**Figure 1:**
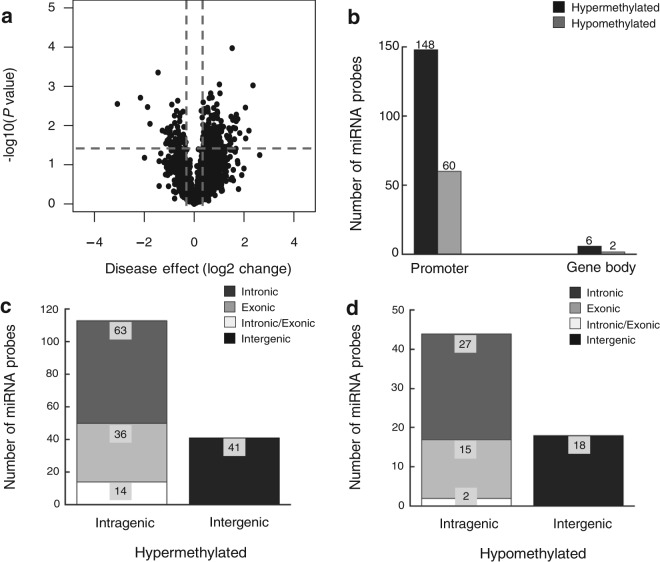
miRNA gene promoters are preferential target sites of aberrant DNA methylation in FECD. **(a)** Volcano plot shows the function of disease effect (log2 (fold change), x-axis) versus the statistical significance of the result (−log 10 (p-value), y-axis) for a total number of 2,227 probes targeting miRNAs genes on the array. Each dot represents an individual probe. Vertical dotted lines represent fold changes of ±1.3, respectively. The horizontal dotted line indicates the p-value cutoff point (0.05; -log10(0.05) = 1.30103). The dots represent 216 selected differentially expressed probes with p-value < 0.05 and |fold-change| > 1.3. **(b)** Number of differentially methylated probes between FECD and control samples (*p*-value < 0.05), grouped by the probe targeting region in related miRNA genes. **(c)** Genome location of hypermethylated miRNA probes relative to the corresponding host genes. **(d)** Genomic location of hypomethylated miRNA probes relative to the corresponding host genes.

We next examined and compared the DNA methylation levels of individual miRNA sequence between FECD cases and controls by comparing single HM450 probe. Of the 2,227 miRNA-associated probes (targeting 463 miRNA genes), 216 probes (targeting 156 miRNA genes) were differentially methylated in the FECD samples (*p* < 0.05; Fig. 1a). Of the 216 probes, the large majority (154 probes; 71%) were hypermethylated in the FECD samples, and a small minority (62 probes; 29%) of sequences were hypomethylated (Fig. 1b). Almost all of the differentially methylated probes (208 probes; 96%) targeted miRNA promoter sequences (148/154 hypermethylated probes and 60/62 hypomethylated probes; Fig. 1b). None of the miRNA probes were significantly differentially methylated with respect to age or sex, suggesting that these parameters are not major drivers of DNA methylation changes in miRNA genes in FECD patients (data not shown). Table 1 shows details on the 20 top ranking differentially methylated miRNA genes in the FECD samples as compared to controls. *miR199-B* was found to be the most extensively hypermethylated miRNA gene and *miR-1182* was the most extensively hypomethylated miRNA gene in FECD samples (Table 1).

**Table 1:** Top 20 most significantly differently methylated miRNAs in FECD, with 18 of them being hypermethylated and 2 being hypomethylated.

Gene	Probe ID	Host genes	Fuchs_Coeff^a^	Fuchs_Pval	Fuchs_Qval
miR-199B	cg13718827	DNM1 (antisense; intronic)	2.3595	***	0.00128
miR-33B	cg04805065	SREBF1 (sense; intronic)	2.1974	*	0.12825
miR-874	cg18251187	KLHL3 (sense; intronic)	2.0571	**	0.00944
miR-1286	cg08221669	RTN4R (sense; intronic)	1.7488	**	0.02761
miR-1306	cg20689730	DGCR8 (sense; exonic)	1.6852	**	0.03932
miR-130A	cg10512089	intergenic	1.6548	*	0.09226
miR-320B1	cg25023761	intergenic	1.5556	**	0.02459
miR-641	cg26620021	AKT2 (sense; intronic)	1.5535	**	0.03169
miR-942	cg19582647	TTF2 (sense; intronic)	1.5362	*	0.82808
miR-499	cg11231913	MYH7B (sense; intronic)	1.5245	***	0.00004
miR-30B	cg25964744	intergenic	1.4986	*	0.75198
miR-184	cg23721598	ANKRD34C-AS1(antisense; intronic)	1.4823	**	0.02005
miR-194-2	cg24803202	MIR194-2HG (sense; exonic)	1.4674	*	0.42089
miR-25	cg22638766	MCM7 (sense; intronic)	1.4593	*	0.13411
miR-662	cg26775123	MSLNL (antisense; exonic)	1.4426	*	0.80350
miR-1471	cg06046580	intergenic	1.3949	*	0.08720
miR-199A1	cg18544365	DNM2 (antisense; intronic)	1.3434	*	0.26307
miR-320D1	cg23483562	intergenic	1.2870	*	0.60071
miR-1182	cg20821842	FAM89A (sense; exonic)	−3.0805	**	0.00667
miR-193A	cg08667128	intergenic	−1.4483	***	0.00030

^a^*P ≤ 0.05

**P ≤ 0.01

***P ≤ 0.001.

### Genomic locations of differentially methylated miRNA genes with respect to their host genes

The majority of miRNAs are located within intronic or exonic regions of protein-coding genes (host genes), and increasing evidence suggests a functional relationship between miRNAs and their host genes^37^. Therefore, we next sought to examine the spatial relationship of the differentially methylated miRNA probes with their host genes. We mapped all 216 differentially methylated miRNA probes to their host genes using the Ensembl Genome Browser (https://uswest.ensembl.org/index.html). Of the 154 hypermethylated miRNA probes, 74% (113 probes) corresponded to intragenic sequences and 27% (41 probes) occurred in intergenic sequences (Fig. 1c). Of the intragenic probes, 41% (63 probes) occurred within intronic sequences, 23% (36 probes) within exonic sequences, and 9% (14 probes) in intron/exon boundaries (Fig. 1c). Similar to the hypermethylated probes, the majority of hypomethylated miRNA probes occurred in intragenic sequences (71%, 44 out of 62 probes) and intronic sequences (44% intronic, 24% exonic, 3% in intron/exon boundary sequences) (Fig. 1d). Together, these data show that the majority of differentially methylated miRNA probes occur in intragenic and intronic sequences of their host genes.

### Gene body regions of miRNA host genes are frequent targets of aberrant DNA methylation in FECD

**Figure 2:**
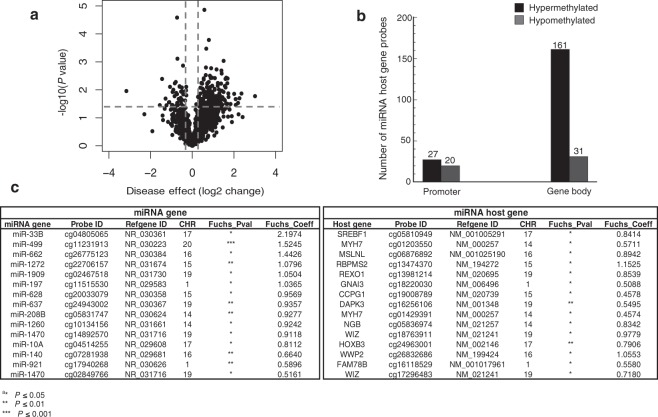
MiRNAs and host genes were co-methylated at corresponding promoter regions in FECD. **(a)** Volcano plot showing the function of disease effect (log2 (fold change), x-axis) versus the statistical significance of the result (−log 10 (p-value), y-axis) for a total number of 1,823 probes targeting miRNAs host genes on the array. Each dot represents a probe. Vertical dotted lines represent fold changes of ±1.3, respectively. The horizontal dotted line indicate the p-value cutoff point (0.05; −log10(0.05) = 1.30103). The dots represent 239 selected differentially expressed miRNA host genes with p-value < 0.05 and |fold-change| > 1.3. **(b)** Number of differentially methylated probes associated with miRNA host genes between FECD and control samples (*p* < 0.05), grouped by the probe targeting region in related miRNA host genes. **(c)** A subset of 15 miRNAs and their host genes with co-methylation at promoter CpG sites in FECD.

Emerging evidence has revealed mutually regulatory roles between particular miRNAs and their host genes^38–41^. Therefore, to decipher the function of DNA methylation in the epigenetic regulation of miRNAs and their host genes, we next assessed the methylation status of host genes for the 156 differentially methylated miRNA genes in FECD. The list of 156 host genes was curated using publicly available miRNA databases, including Ensembl Genome Browser (https://uswest.ensembl.org/index.html), miRIAD (http://www.miriad-database.org), and miRStart (http://mirstart.mbc.nctu.edu.tw/about.php). A total of 1,823 probes mapped to CpG sites in the set of 156 host genes. A volcano plot display of the DNA methylation status of the 1,823 probes showed that 239 probes were differentially methylated in FECD samples compared to control samples (*p* < 0.05; Fig. 2a). In fact, most (188; 79%) of the 239 differentially methylated CpG sites were hypermethylated in FECD samples, with only a minority (51; 21%) of them hypomethylated. Alignment of the probe sequences to the human genome database revealed that the vast majority (192; 80%) of the 239 differentially methylated CpG sites were located in the gene body regions of miRNA host genes (Fig. 2b). A substantial proportion (161; 86%) of the 188 hypermethylated probes targeted CpG sites within gene bodies, whereas only 14% of them were mapped to the promoter regions of corresponding miRNA host genes. Similarly, a high percentage (31; 61%) of the 51 hypomethylated probes also mapped to gene body sequences of miRNA host genes. Table 2 provides detailed information on the top 20 differentially methylated miRNA host genes identified in the FECD samples as compared to controls. Given that miRNAs may be co-regulated with their host genes, we further analyzed the DNA methylation data for co-methylation patterns. We identified a subset of miRNAs and their host genes to be co-methylated at their corresponding promoter CpG sites, suggesting that DNA methylation may play an important role in regulating the co-expression of miRNAs and their host genes in FECD (Fig. 2c).

**Table 2:** List of top 20 methylated miRNA host genes in FECD, with 14 of them being hypermethylated and 6 being hypomethylated.

Gene	Probe ID	Resident miRNA	Fuchs_Coeff^a^	Fuchs_Pval	Fuchs_Qval
CIT	cg03339668	miR-1178 (sense; exonic)	3.0116	*	0.13416
SREBF1	cg09796270	miR-33B (sense; intronic)	2.3618	*	0.08757
C9orf3	cg21189849	miR-23B (sense; intronic)	2.2652	*	0.18803
SLIT2	cg19940312	miR-218-1 (sense; intronic)	2.1981	*	0.36809
WHSC2	cg00248861	miR-943 (sense; exonic)	2.0870	*	0.52318
WWP2	cg26736200	miR-1205 (sense; intronic)	2.0818	*	0.21430
MCM7	cg22420044	miR-25 (sense; intronic)	1.6960	**	0.00692
BCAR3	cg17274827	miR-760 (antisense; intronic)	1.6260	*	0.40336
AKT2	cg15701203	miR-641 (sense; intronic)	1.6229	*	0.80941
DNM3	cg06267617	miR-199A1 (antisense; intronic)	1.5988	**	0.03039
AATK	cg16067628	miR-657 (sense; intronic)	1.5947	**	0.00751
LRP1	cg20029881	miR-1228 (sense; intronic)	1.5654	*	0.26971
HOXB3	cg12910797	miR-10A (sense; intronic)	1.5616	**	0.02261
GNAI3	cg08644463	miR-197 (sense; exonic)	1.2895	*	0.60953
MSLNL	cg02266878	miR-662 (antisense; exonic)	−1.2366	*	0.56317
FBXL18	cg09554310	miR-589 (sense; intronic)	−1.2813	*	0.19659
WDR82	cg12048331	miR-LET7G (sense; intronic)	−1.3231	*	0.91190
CCPG1	cg12392104	miR-628 (sense; intronic)	−1.4477	**	0.00787
RUNX1	cg00291213	miR-802 (antisense; intronic)	−1.5569	*	0.55194
PVT1	cg02901522	miR-1205 (sense; intronic)	−3.1615	*	0.05741

^a^*P ≤ 0.05

**P ≤ 0.01

***P ≤ 0.001.

### Validation of miRNA DNA methylation changes using MethyLight

**Figure 3:**
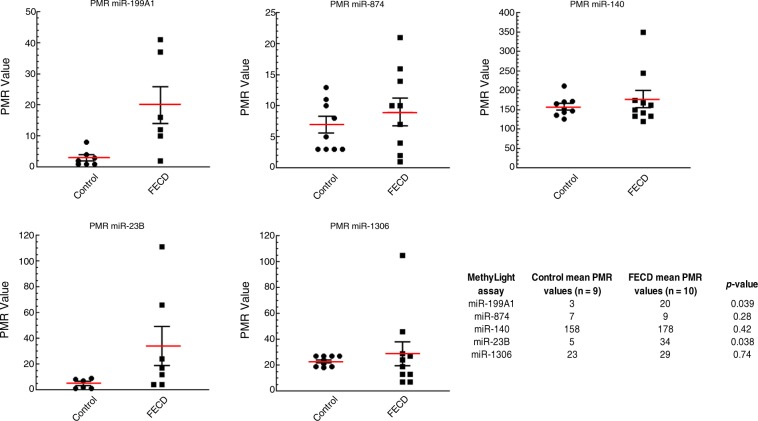
MethyLight analysis for *miR-199A1*, *miR-874*, *miR-140*, *miR-23B* and *miR-1306* in FECD and control endothelial tissues. The methylation levels of five miRNA genes were quantified by real-time PCR-based MethyLight assays on samples from control (n = 9) and FECD (n = 10) samples. MethyLight data are presented as percent of methylated reference (PMR). The table lists the mean PMR values for the control and FECD samples and p-values for each MethyLight assay.

To validate and quantify miRNA DNA hypermethylation changes identified by the global array^30^, MethyLight analysis was performed on an additional cohort of control and FECD patient corneal samples (Supplementary Table 2)^42^. We assessed the promoter DNA methylation status of following miRNAs: *miR-199A1, miR-874, miR-140, miR-23B, and miR-1306* **(**Supplementary Fig. 2, Supplementary Table 1). These miRNAs were selected for validation because of their important roles in the pathogenesis of FECD or in the regulation of key cellular processes (e.g. cell survival, oxidative stress, inflammation, fibrosis, and deposition of extracellular matrix)^43–50^. The MethyLight results confirmed DNA hypermethylation in the FECD samples compared with control samples (Fig. 3) and verified our array findings. All five MethyLight assays gave higher mean DNA methylation values (Percent of Methylated Reference, PMR) in the FECD samples compared to the control samples. Among these five miRNA genes, *miR-199A1* and *miR-23B* were found to be methylated in the FECD samples but unmethylated in the control samples (Fig. 3). For *miR-199A1*, the average PMR values were 20 and 3 for FECD and control samples, respectively (*p* = 0.039; Fig. 3). For *miR-23B*, the mean PMR values were 34 for FECD samples and 5 for control samples (*p* = 0.038; Fig. 3). There are considerable inter-individual variations in DNA methylation levels of each miRNA among FECD patients, as reflected by PMR values (Fig. 3). We observed higher DNA methylation levels of miRNAs in patients with a more severe disease state. Recent studies have demonstrated that DNA methylation levels can be associated with disease severity^51,52^. Taken together, these MethyLight results confirmed hypermethylation of miRNA sequences in FECD tissue compared with normal control samples found in the genome-wide array using an independent set of patient samples.

### Relationship between miRNA DNA methylation status and expression

**Figure 4:**
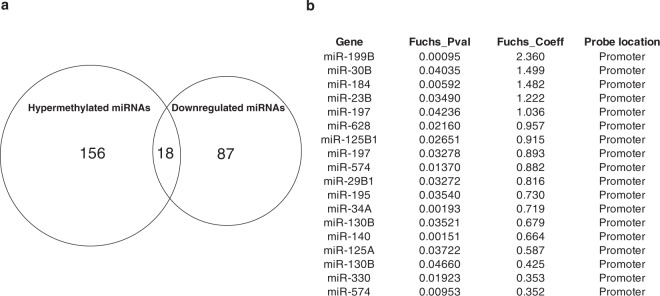
Differentially methylated and expressed miRNAs between FECD and donor samples. **(a)** Venn diagram showing the overlap of differentially methylated miRNA genes (n = 156) and differentially expressed miRNA genes (n = 87) between FECD and control samples. **(b)** List of the 18 miRNAs that have concurrent hypermethylation and decreased expression in FECD compared to the control samples.

The majority of the miRNA DNA methylation changes observed in FECD tissues occurred in miRNA promoter sequences. Because promoter DNA methylation is often inversely correlated with gene expression levels^53,54^, we next sought to determine how DNA methylation might affect miRNA expression in FECD tissue. Matthaei *et al*. previously compared miRNA expression profiles of corneal endothelial samples obtained from FECD patients and from normal donors using transcriptome analysis^36^. Their results demonstrated downregulation of 87 miRNAs in FECD compared with normal endothelium and suggested that altered miRNA expression may play an important role in the pathogenesis of FECD disease^36^. Therefore, we integrated our DNA methylation data with their miRNA expression data and generated a Venn diagram showing all differentially methylated and differentially expressed miRNAs (Fig. 4a). Of 156 miRNAs that are hypermethylated and 87 miRNAs that have down-regulated expression in FECD, 18 miRNAs have concurrent hypermethylation and decreased expression in FECD compared to the control samples (Fig. 4a,b). In particular, miR-199b-5p expression was almost completely silenced^36^ and it was the miRNA with the highest level of promoter hypermethylation (Fig. 4b). This strong correlation between down-regulated miR-199b-5p expression and its high level of promoter hypermethylation in FECD suggests that miR-199b-5p directed pathways may have an important role in FECD pathogenesis.

### miR-199b-5p negatively regulates Snai1 and ZEB1 expression in corneal endothelial cells

**Figure 5:**
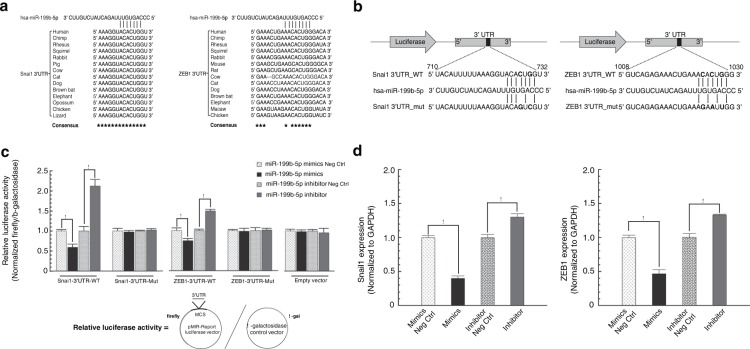
miR-199b-5p binds directly to the 3′-UTR of Snai1 and ZEB1 mRNAs. **(a)** *In silico* analyses predicted a highly conserved binding site for miR-199b-5p in the 3′-UTR of Snai1 and ZEB1 mRNAs across species. **(b)** The luciferase reporter plasmids containing the wild-type 3′-UTR or mutant 3′-UTR of Snail and ZEB1 with the putative binding sites for miR-199b-5p. **(c)** The direct binding of miR-199b-5p to 3′-UTR of Snai1 and ZEB1 mRNAs was detected by a dual luciferase activity assay. The luciferase reporter plasmids and β-galactosidase expression plasmids were co-transfected into HEK293 or HCEnC-21T cells with either miR-199b-5p mimic, miR-199b-5p mimic negative control (Neg Ctrl), miR-199b-5p inhibitor, and miR-199b-5p inhibitor negative control (Neg Ctrl). Luciferase activity was measured by the dual luciferase reporter assay system. Relative luciferase activities were calculated by normalizing firefly luciferase activity to β-galactosidase activity in the same sample to correct for transfection efficiencies. Data are represented as the mean ± SEM (n = 3; *p* < 0.05). **(d)** The effect of miR-199b-5p on the expression of Snail and ZEB1. HCEnC-21T cells were transfected with miR-199b-5p mimic or miR-199b-5p inhibitor with their corresponding negative controls. 48 hours post-transfection, total RNA was extracted and mRNA expression levels of Snail and ZEB1 were quantified by qRT-PCR. Data are represented as the mean ± SEM (n = 3; *p* < 0.05).

MiRNAs can negatively regulate gene expression by directly binding to specific sequences in the 3′-UTR of target mRNAs and inducing mRNA cleavage or translation inhibition^55^. In mammals, there is a high-degree of Watson-Crick base-pairing between miRNA and target mRNA at nucleotides 2–7 at the 5′ end of miRNA, termed the “seed match”^56^. Mismatches in the miRNA-mRNA duplex were found to be ineffective in repressing gene expression^57^. To further delineate the functional role of miR-199b-5p in FECD pathogenesis, we performed *in silico* analysis to predict putative target genes and corresponding binding sites using two computational prediction algorithms (Targetscan and miRmap). More than one thousand targets of miR-199b-5p were predicted from these programs. Snai1 and ZEB1 were of particular interest because their overexpression leads to excessive extracellular matrix production in FECD^58^. Both prediction tools independently gave Snai1 and ZEB1 high scores (97 and 83.4 respectively). Sequence alignment analyses revealed a highly conserved miRNA-199b-5p binding motif in the 3′-UTR of both Snai1 and ZEB1 across many species (Fig. 5a). In particular, this predicted binding site was located in the 3′-UTR of human Snai1 (positions 725–731; NM_005985.3) and ZEB1 (positions 1023–1029; NM_001128128.2) (Fig. 5b).

To investigate whether Snai1 and ZEB1 are direct targets of miR-199b-5p and to assess the role of miR-199b-5p in regulating Snai1 and ZEB1 expression in corneal endothelial cells, we cloned human Snai1 and ZEB1 3′-UTR sequences into a luciferase reporter vector (pMIR-Snai1-WT and pMIR-ZEB1-WT; Fig. 5b,c). We additionally cloned fragments of human Snai1 and ZEB1 3′-UTR with mutated miR-199b-5p binding sites into a luciferase reporter vector (pMIR-SNAI1-Mut and pMIR-ZEB1-Mut; Fig. 5b,c). The reporter plasmids (pMIR-control, pMIR-Snai1/ZEB1-WT, and pMIR-Snai1/ZEB1-Mut) were co-transfected into human corneal endothelial cells or HEK293 cells with either miR-199b-5p mimics, miR-199b-5p mimic-negative control, miR-199b-5p inhibitor, or miR-199b-5p inhibitor-negative control, along with a β-galactosidase expression plasmid as an internal control. The dual-luciferase reporter assays showed that miR-199b-5p regulated Snai1 and ZEB1 by binding directly to their 3′-UTR sequences. MiR-199b-5p mimics significantly decreased the luciferase activity approximately 50% (*p* = 0.039) and 30% (*p* = 0.009), respectively, in cells co-transfected with pMIR-Snai1-WT and pMIR-ZEB1-WT, compared with their corresponding negative controls (Fig. 5c). In contrast, miR-199b-5p inhibitor significantly increased their luciferase activities by 2.1- (*p* = 0.005) and 1.5-fold (*p* = 0.04), respectively (Fig. 5c). However, miR-199b-5p mimic and inhibitor had no effect on luciferase activities in cells co-transfected with pMIR-Snai1-Mut, pMIR-ZEB1-Mut, or empty pMIR-control vectors *(p* > 0.05*;* Fig. 5c). Taken together, these data show that miR-199b-5p directly targets the 3′-UTRs of Snai1 and ZEB1 mRNA transcripts.

To further evaluate the effect of miR-199b-5p on Snai1 and ZEB1 expression in human corneal endothelial cells, we transfected miR-199b-5p mimic, inhibitor, or negative controls into human corneal endothelial cells and measured the expression levels of Snai1 and ZEB1 by qRT-PCR. We found that the miR-199b-5p mimic significantly inhibited Snai1 and ZEB1 expression by 50%, compared to negative control group (*p* = 0.0002 and *p* = 0.002*, respectively*; Fig. 5d). In contrast, the miR-199b-5p inhibitor had the opposite effect and resulted in increased Snai1 and ZEB1 expression (~1.3 fold, *p* = 0.012 and *p* = 0.005*, respectively*; Fig. 5d). These results demonstrate that miR-199b-5p can directly bind to and negatively regulate Snai1 and ZEB1 in human corneal endothelial cells.

## Discussion

FECD is the most common type of corneal endothelial dystrophy and a leading indication for corneal transplantation in patients in the United States^6,59^. We previously identified global DNA methylation changes that occur in the corneal endothelial tissue of FECD patients and specifically observed a high number of DNA methylation alterations occurring in miRNA sequences^30^. This finding was intriguing because prior reports had demonstrated that miRNAs were differentially expressed in the corneal endothelium during aging^60^, and that widespread downregulation of miRNA levels occurred in the corneal endothelium of patients with late-onset FECD^36,61^. Because DNA methylation has been shown to be a mechanism for regulating miRNA expression^33^, we performed a sub-analysis of the miRNA DNA methylation array data. The most differentially methylated miRNA sequences were further validated by quantitative MethyLight assay using an additional patient cohort. *MiR-199B* was identified as the most extensively hypermethylated miRNA sequence in FECD and was selected for additional analysis because its expression was almost completely silenced in FECD^36^. *In silico* analyses identified Snai1 and ZEB1 as potential direct targets of miR-199b-5p. Using a luciferase reporter assay, we confirmed that miR-199b-5p directly targeted the 3′-UTR of both Snai1 and ZEB1 transcripts and negatively regulated their expression. Collectively, these results demonstrate that miR-199b-5p hypermethylation may contribute to late-onset FECD pathogenesis. Our findings suggest that miR-199b-5p hypermethylation leads to its down-regulated expression and consequently results in the increased expression of miR-199b-5p target genes, including Snai1 and ZEB1.

MiRNAs are small non-coding RNAs that negatively regulate gene expression by binding to specific sequences in the 3′-UTR of target mRNAs^60,62^. Such interactions may result in either translation inhibition or induction of mRNA cleavage^62^. Numerous studies have shown that miRNAs are evolutionarily conserved and are key regulators of diverse biological processes such as development, cell proliferation and differentiation, apoptosis and metabolism^63^. MiRNAs also have important regulatory roles in disease progression, including oncogenesis^64,65^. The molecular mechanisms that control miRNA expression are therefore of critical importance in better understanding normal physiologic processes and disease pathogenesis. Recently, DNA methylation has emerged as a key regulatory mechanism of miRNA expression in several different tissues and disease states^64–67^.

In this study, we have demonstrated aberrant DNA methylation of miRNA sequences in corneal endothelial tissue of FECD patients. Our array dataset included 2,227 probes associated with 463 miRNA genes, with multiple probes targeting single miRNA genes. We identified 216 probes associated with 156 miRNA genes that were differentially methylated between FECD and control samples, and the vast majority were hypermethylated in FECD. Furthermore, we found that the aberrant DNA methylation occurred almost exclusively in the promoter regions of miRNAs. Since promoter methylation and gene expression are usually inversely correlated, these results suggest DNA hypermethylation as a potential mechanism for the widespread downregulation of miRNA levels in FECD^36^. This preferential hypermethylation of miRNA gene promoters was also reported in other studies^33,68–70^.

To further investigate the methylated probes in a broader genomic context, we mapped the 50 bp sequences of all 216 differentially methylation probes associated with 156 miRNA genes to the human genome. We found that approximately three-quarters of these probes were located within introns and/or exons of relevant host genes. The intragenic resident miRNAs on the same strand as their host genes can be co-transcribed by RNA polymerase II and co-regulated with their host genes^71^. A genome-scale DNA methylation analysis specifically on miRNA host genes revealed that miRNA host genes were frequent targets for aberrant DNA methylation and in particular downregulation of miR-10a was correlated with the promoter hypermethylation of its host gene *HOXB4* in tumorigenesis^39^. Our data found that the miRNA host genes were differentially methylated in FECD and that their gene body regions were preferential targets of aberrant methylation. Even though gene body methylation is positively correlated with gene expression^72^, we were unable to measure the changes in mRNA levels of miRNA host genes on the same sample cohorts used in the DNA methylation analyses because of the low cellular yield. Therefore, the physiological relevance of DNA methylation changes of miRNA host genes in FECD remains to be further explored. Additionally, a subset of miRNA genes and their host genes shared hypermethylation of their individual promoters, suggesting that DNA methylation may play an important role in repressing the expression of certain miRNAs and their host genes simultaneously in FECD.

Using two independent DNA methylation assay technologies and two separate patient cohorts, we identified miR-199B as the most extensively hypermethylated miRNA in the FECD samples. Interestingly, miR-199b-5p has been shown to be almost completely silenced in FECD tissues^36^. We were unable to perform side-by-side comparative miRNA transcriptome analysis on the same sample cohorts used in the DNA methylation analyses because of the low cellular yield from the FECD samples. To delineate the mechanism by which miR-199b-5p may contribute to FECD pathogenesis, we used computational algorithms to search for putative target genes, and identified Snai1 and ZEB1 as having high prediction scores. Further functional analyses using a luciferase reporter assay confirmed both 3′-UTRs of Snai1 and ZEB1 transcripts as being direct targets of miR-199b-5p. Our result is consistent with the prior finding that miR-199a-5p, a close family member of miR-199b-5p, directly binds the 3′-UTR of the Snai1 mRNA and reduces Snai1 protein level via the UGUGACC motif in its seed sequence^73^. Members of the same miRNA family can have similar physiological function and share the same predicted targets because of their conserved sequence and structural configuration^74^. Our finding that the 3′-UTRs of Snai1 and ZEB1 have the same predicted target site recognized by the identical seed sequence for both miR-199a-5p and miR-199b-5p supports the miR-199 family as having an important regulatory role in Snai1 and ZEB1 expression and function.

Snai1 and ZEB1 are zinc finger transcription factors that regulate gene expression in multiple tissues, including the cornea. Okumura *et al*. showed that immortalized corneal endothelial cells obtained from late-onset FECD patients highly expressed Snai1 and ZEB1 had excessive production of ECM proteins, including type I collagen and fibronectin^58^. Katikireddy *et al*. found that Snai1 expression level is significantly upregulated in *ex vivo* FECD specimens as compared to control samples^75^.

A phenotypic clinical feature of FECD is the development of corneal guttae, which are abnormal collagenous excrescences of the corneal endothelial basement membrane (Descemet’s membrane). Recent studies have shown that Snail and ZEB1 can also reduce cell adhesion, increase cell migratory capacity^76–78^, and promote apoptosis^79,80^. These phenotypic features have also been observed during FECD pathogenesis^81–83^.

**Figure 6:**
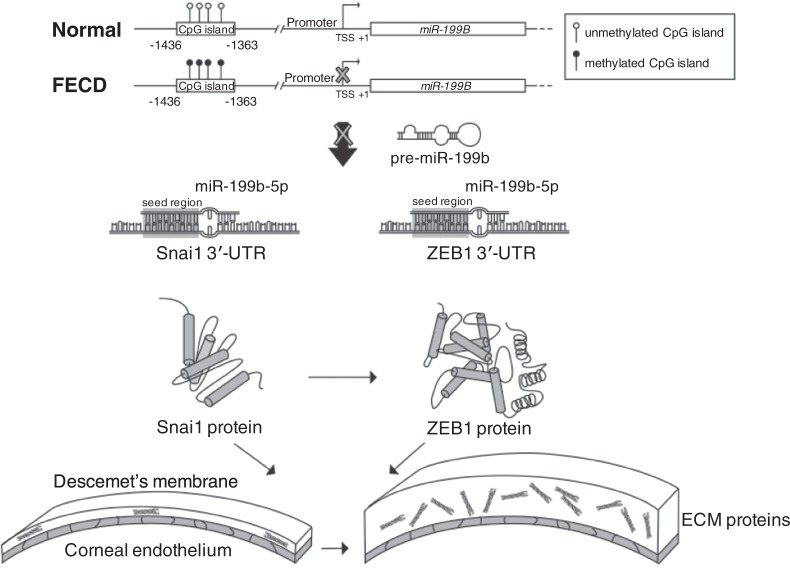
*MiR-199B* is hypermethylated in CpG island at its promoter region and its mature transcript miR-199b-5p directly inhibits the expression of Snai1 and ZEB1 in FECD. *MiR-199B* is the most extensively hypermethylated miRNA gene in FECD. Its mature transcript miR-199b-5p functions as a direct negative regulator of two zinc finger transcription factors, Snai1 and ZEB1, which have been shown to lead to increased production of extracellular matrix proteins in FECD.

Our findings support a model in which aberrant promoter hypermethylation of miR-199b-5p in FECD leads to the up-regulated expression of Snai1 and ZEB1 expression and consequent pathologic overproduction of ECM proteins in the cornea (Fig. 6). Dysregulated DNA methylation of miRNA promoters has been found to be a biomarker in the detection, diagnosis, and prognosis of various cancers types including breast^84^, gastrointestinal^85^, and lung^86^. Our results provide a novel mechanistic insight into the function of DNA methylation in the pathogenesis of FECD and support further studies to determine how methylation of miR-199b-5p may be used as a clinical biomarker of phenotype expression in FECD. In the past few years, there have been major advances in testing blood, saliva, and cheek swab samples to genetically screen for corneal dystrophies^87,88^. Further studies are needed to determine if DNA methylation changes can be detected in these tissue samples that correspond to corneal methylation changes. Anterior chamber paracentesis samples can potentially be assayed in the future as another alternative. A recent study has shown that DNA methylation changes associated with bladder cancer are currently being screened for in urine samples^89^.

The epigenome is increasingly being recognized as fertile ground for drug development, as DNA methylation is dynamic and pharmacologically reversible^90^. Several drugs currently exist that target and inhibit DNA methylation^90^. Of these, the cytidine analogues 5-azacytidine (5-Aza-CR) and 5-aza-2′-deoxycytidine (5-Aza-CdR) are the two most potent DNMT inhibitors and have been approved by the Food and Drug Administration (FDA) in the USA for the treatment of myeloid malignancies and other solid tumors^91–93^. A large number (>30) of different, epigenome-targeting drugs are currently in clinical trials^94^. Our results support further studies to test the demethylation and remethylation effect of 5-Aza-CdR on normal and diseased (FECD) corneal endothelial cells.

## Materials and Methods

### Ethical compliance

Institutional Review Board (IRB)/Ethics Committee approval was obtained from the University of California, San Francisco Human Research Protection Program (Study Number 11–07020). Written informed consent was obtained from all participants. Protected health information was masked according to HIPAA privacy standards and the patient database was managed securely in Research Electronic Data Capture (REDCap)^95^. All of the described research adheres to the tenets of the Declaration of Helsinki.

### Subjects and selection criteria

Corneal endothelium was collected from FECD patients undergoing endothelial keratoplasty by two surgeons (D.G.H. and J.R.R-N.) at the University of California, San Francisco. Patients with a diagnosis of FECD and scheduled for endothelial keratoplasty between the dates of 2/12/2013 and 10/27/2014 (for Illumina Infinium HumanMethylation450 BeadChip analysis) and 1/18/2017 and 1/29/2018 (for MethyLight analysis) were recruited for the study. Written consent was obtained from participating patients and clinical information from the most recent office visit was collected from electronic medical documentation, including guttata score and pachymetry. Age- and gender-matched non-FECD corneal endothelial samples were obtained from an eye bank (SightLife, Seattle WA; and San Diego Eye Bank, San Diego CA) and processed in the same manner as the FECD samples.

### DNA methylation microarray

Array-based DNA methylation data was collected in our prior studies^30^. The IDAT files are available on the GEO DataSets database [accession number GSE94462; National Center for Biotechnology Information (NCBI), Bethesda, MD, USA].

### MethyLight assay

DNA methylation levels were measured using MethyLight technology, which is a quantitative, TaqMan-based real-time PCR assay using bisulfite converted DNA as a template^96^. Genomic DNA (200–500 ng) for each sample was converted with bisulfite using the Zymo EZ DNA Methylation kit (Zymo Research, Irvine, CA) as per the manufacturer’s instructions. M. SssI-treated DNA sample was included as a methylated reference. An interspersed *ALU* repeats-based methylation-independent reaction was also included as a normalization control. The percent of methylated reference (PMR) values for each sample were calculated for each sample as follows: PMR = 100 × (GENE/ALU)_sample_/(GENE/ALU)_M. SssI-Reference_. The following probes targeting miRNA promoter sequences were chosen for validation with the MethyLight assay because they were significantly differentially methylated in FECD samples compared to the control samples: miR-199A1 (cg18544365), miR-874 (cg18251187), miR-140 (cg07281938), miR-23B promoter (cg00351472), and miR-1306 (cg20689730). A complete list of primers and probes for all MethyLight reactions is provided in Supplementary Table 1.

### Construction of the luciferase reporter plasmids

The putative binding sites in the 3′-UTRs of *Snai1* and *ZEB1* genes were bioinformatically predicted for miR-199b-5p using multiple computational prediction algorithms, including TargetScan and miRmap. The 3′-UTR sequences of both genes were then amplified from genomic DNA obtained from HEK293 cells with Phusion® High-Fidelity DNA Polymerases (NEB, Ipswich, MA) and cloned into the multiple cloning site of the pMIR-REPORT luciferase miRNA expression vector (Thermo Fisher Scientific, Waltham, MA) using the In-fusion HD Cloning Kit (Clontech, Mountain View, CA). The primers used to amplify the *Snail* 3′-UTR were: 5′-GAGTGATGAAAGCTGCGCACTAGTGGCAATTTAACAATGTCTGAAAAGG-3′, and 5′-AAAGATCCTTTATTAAGCTTCTGTACATATAACTATACAAAACGTTTCC-3′; the primers used to amplify the *ZEB1* 3′-UTR were: 5′-GAGTGATGAAAGCTGCGCACTAGTGCAGGGA CTAACAATGTTAATCTG-3′, and 5′-AAAGATCCTTTATTAAGCTTCTACAGTCCAAGGC AAGTATAAATG-3′. Two mutant Snai1 and ZEB1 3′-UTR reporter vectors that lacked the binding sites for miR-199b-5p were generated using standard PCR-based overlap-extension protocols. The primers used to amplify the mutated *Snai1*3′-UTR were: *Snai1* 3′-UTR-M1, 5′-GAGTGATGAAA GCTGCGCACTAGTGGCAATTTAACAATGTCTGAAAAGG-3′, and 5′-AATACGACTGTACCTTTAAAAATGTAAAC-3′; *Snai1* 3′-UTR-M2, 5′-AAGGTACAGTCG TATTTATATTTCAAAC-3′, and 5′-AAAGATCCTTTATTAAGCTTCTGTACATATAACTAT ACAAAACGTTTCC-3′. The primers used to amplify the mutated *ZEB1* 3′-UTR were: *ZEB1* 3′-UTR-M1, 5′-GAGTGATGAAAGCTGCGCACTAGTGCAGGGACTAACAATGTTAATCTG-3′, and 5′-AT GTCCAATTCTTTCAGTTTCTCTGACAGAGTCAGT-3′; *ZEB1* 3′-UTR-M2, 5′-ACTGAAAGAATTGGACATTTCATCCTTCAATTCCTCGG-3′, and 5′-AAAGATCCTTTAT TAAGCTTCTACAGTCCAAGGCAAGTATAAATG-3′. All clones were verified by DNA sequencing (Elim Biopharmaceuticals, Hayward, CA).

### Dual luciferase reporter assay

Dual luciferase reporter assays were performed to validate *Snail1* and *ZEB1* as *bona fide* miRNA target genes. Briefly, 0.3 × 10^6^ of HCEnC-21T cells^97^ or HEK293 cells were seeded in 24-well plates and then co-transfected with 500 ng of pMIR-REPORT wild-type or mutant plasmid, 100 ng of β-gal plasmid, and 25 nmol miR-199b-5p mimic, 25 nmol scrambled mimic negative control, 50 nmol miR-199b-5p inhibitor, or 50 nmol scrambled inhibitor negative control (Life Technologies, Carlsbad, CA), using Lipofectamine 3000 (Life Technologies, Carlsbad, CA) in OptiMEM (Gibco, CA). A β-galactosidase expression plasmid was used as an internal control for transfection efficiency. Forty-eight hours after transfection, cells were subjected to lysis and firefly luciferase and β-galactosidase enzymatic activities were measured consecutively using a dual-luciferase reporter assay system (Applied Biosystems, Bedford, MA) as per the manufacturer’s instructions. Relative firefly luciferase activity (firefly luciferase activity/β-galactosidase enzymatic activities) were expressed as changes relative to that value of the negative control, which was set as 1. Three independent experiments were performed in triplicate.

### Quantitative real-time PCR (qRT-PCR)

HCEnC-21T cells were seeded in 24-well plates (0.3 × 10^6^ cells/well) and then transfected with 50 nmol miR-199–5p mimic, scrambled mimic negative control, miR-199b-5p inhibitor or scrambled inhibitor negative control using Lipofectamine RNAiMAX (Life Technologies, Carlsbad, CA). Forty-eight hours after transfection, cells were lysed and total RNA was extracted using PureLink^TM^ RNA mini Kit (Ambion, Foster City, CA). RNA concentration was measured on a NanoDrop Spectrophotometer (ThermoFisher Scientific, Wilmington, DE). Superscript III reverse transcriptase (Invitrogen) was used to generate single-stranded cDNA from 0.5 μg of total RNA with oligo dT, as per the manufacturer’s instructions. qRT-PCR was run with SYBR green PCR master mix (ThermoFisher Scientific, Wilmington, DE) using ABI Prism 7000 Real-Time PCR Detection System (Applied Biosystems, Bedford, MA). mRNA transcript abundances were determined using specific primers as follows: 1) Snai1: 5′-GACCCACACTGGCGAGAAGC-3′ and 5′-GCCTGGCACTGGTACTTCTTGACATC-3′; 2) ZEB1: 5′-GCTGGGAGGATGACACA GGAAAGG-3′ and 5′-GGTCCTCTTCAGGTGCCTCAGG-3′; 3) GAPDH: 5′-CCATCTTCCAG GAGCGAGATCCCTC-3′ and 5′-CTGCAAATGAGCCCCAGCCTTC-3′. All samples were run in triplicate. All qRT-PCR reactions were run as follows: 2 min at 50 °C, 10 min at 95 °C, 15 s at 95 °C, and 1 min at 60 °C (40 cycles) with a mixture containing 1 μl of cDNA template, 7.5 μl qPCR master mix and 266.7 nmol l^−1^ of each primer in a total volume of 15 μl (ThermoFisher Scientific, Wilmington, DE), as per the manufacturer’s instructions. Data were collected during the 1 min − 60 °C extension step. Melt curves were performed using the following program: 15 s at 95 °C, 2 min at 60 °C, and 15 s at 95 °C with a step of 0.5 °C every cycle. Melting curve analyses showed no primer-dimers or non-specific products. Data are presented as fold change in gene expression normalized to GAPDH. Relative quantification of expression was calculated with the 2^−ΔΔCt^ method^98^.

### Statistical analyses

Statistical analyses on genome-wide methylation data of miRNA genes were executed in R^30^. All other analyses were performed using two-tailed t-test or alternative non-parametric Wilcoxon test to compare mean values using R software. Differences with p-values less than 0.05 were considered statistically significant and the corresponding p values were indicated.

## Supplementary information

Aberrant DNA methylation of miRNAs in Fuchs endothelial corneal dystrophy
