# Author Correction: Aberrant DNA methylation of miRNAs in Fuchs endothelial corneal dystrophy

**DOI:** 10.1038/s41598-020-59304-9

**Published:** 2020-02-06

**Authors:** Peipei Pan, Daniel J. Weisenberger, Siyu Zheng, Marie Wolf, David G. Hwang, Jennifer R. Rose-Nussbaumer, Ula V. Jurkunas, Matilda F. Chan

**Affiliations:** 1 Department of Ophthalmology, University of California, San Francisco, San Francisco, CA USA; 2 Department of Biochemistry and Molecular Medicine, University of Southern California, Los Angeles, CA USA; 3 Francis I. Proctor Foundation, University of California, San Francisco, CA USA; 4 Department of Ophthalmology, Harvard Medical School, and Schepens Eye Research Institute, Massachusetts Eye and Ear, Boston, MA USA

Correction to: *Scientific Reports* 10.1038/s41598-019-52727-z, published online 08 November 2019

The Acknowledgements section in this Article is incomplete.

“We thank Emily Khuc and Selene M. Clay for collecting patients samples for the original genome-wide DNA methylation assay.”

should read:

“We thank Emily Khuc and Selene M. Clay for collecting patients samples for the original genome-wide DNA methylation assay. This study was supported by funds from a Research to Prevent Blindness (RPB) Physician-Scientist Award (to MFC, https://www.rpbusa.org/rpb/), an RPB Unrestricted Grant (to UCSF Department of Ophthalmology, https://www.rpbusa.org/rpb/), the National Institutes of Health (R01 EY022739 to MFC, NIH-NEI EY002162 - Core Grant for Vision Research, NIH/NCRR UCSF-CTSI Grant Number UL1 RR024131 to MFC), and That Man May See (http://thatmanmaysee.org/).”

