# Supplementary material for: Aberrant DNA methylation of miRNAs in Fuchs endothelial corneal dystrophy
Source: Sci Rep. 2019 Nov 8;9:16385. doi: 10.1038/s41598-019-52727-z (PMC6841734; doi:10.1038/s41598-019-52727-z)
Supplement: Supplementary file 1 — Aberrant DNA methylation of miRNAs in Fuchs endothelial corneal dystrophy [file 41598_2019_52727_MOESM1_ESM.pdf]

SUPPLEMENTARY INFORMATION

Aberrant DNA methylation of miRNAs in Fuchs endothelial corneal dystrophy

Peipei Pan[1], Daniel J. Weisenberger[2], Siyu Zheng[1], Marie Wolf[1], David G. Hwang[1,3], *Jennifer* R. *Rose-Nussbaumer*[1,3], Ula V. Jurkunas[4], Matilda F. Chan[1,3]

[1]Department of Ophthalmology, University of California San Francisco School of Medicine, CA, USA
[2]Department of Biochemistry and Molecular Medicine, University of Southern California, Los Angeles, CA, USA
[3]Francis I. Proctor Foundation, University of California, San Francisco, CA, USA
[4]Department of Ophthalmology, Harvard Medical School, and Schepens Eye Research Institute, Massachusetts Eye and Ear, Boston, MA.
*Correspondence and requests for materials should be addressed to Matilda F. Chan (email: matilda.chan@ucsf.edu)

Conflict-of-interest notification statement: DJW is a consultant for Zymo Research Corporation, Irvine, CA

Supplementary Figure 1.

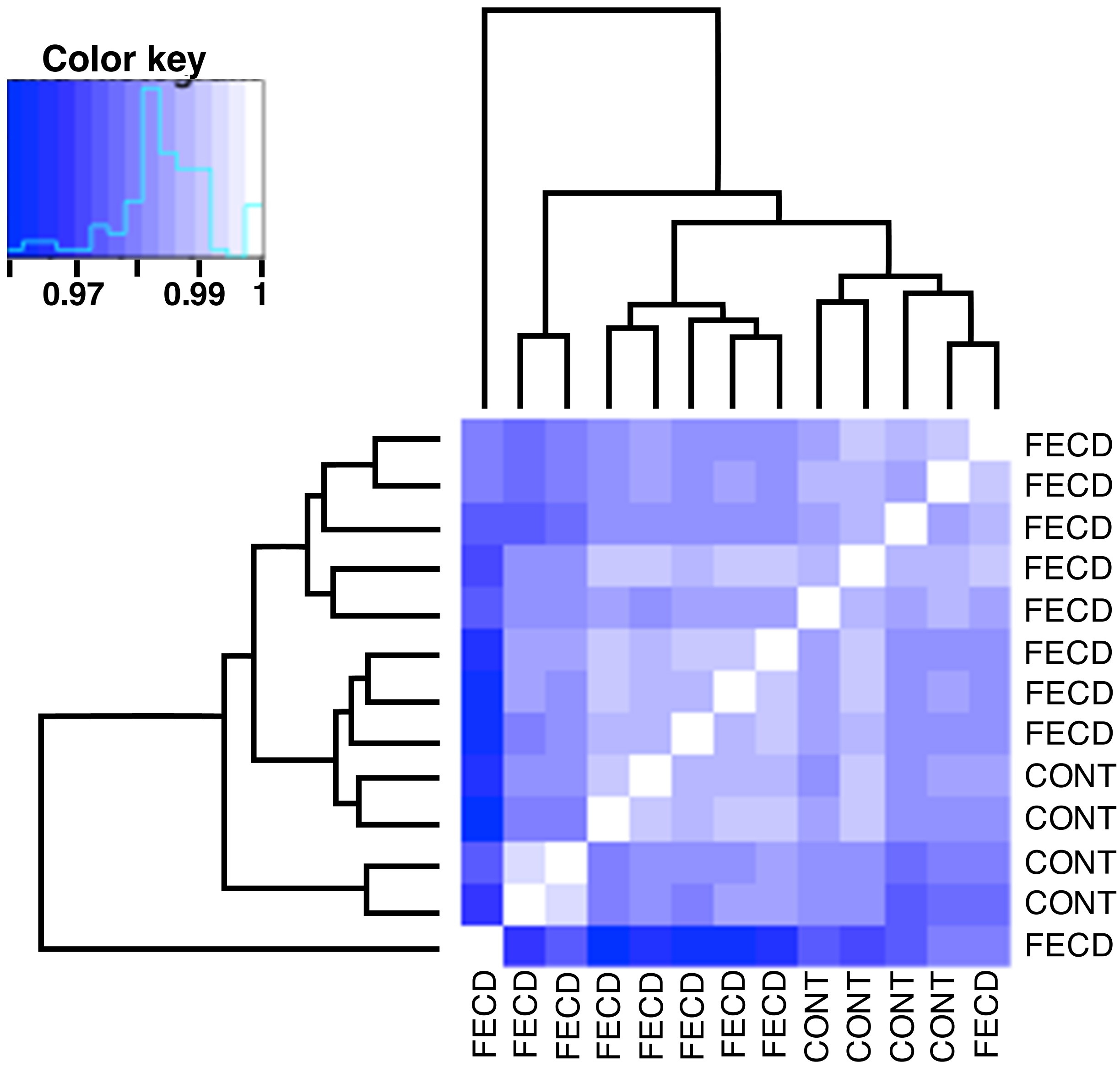

Supplementary Figure S1. Heatmap visualization of pairwise Kendall rank correlation coefficients comparing miRNA gene methylation pattern between FECD and control corneal endothelium samples with dendrogram to show clustering (Euclidean distance). Control and FECD samples separate according to disease variable, with the exception of one FECD sample.

Supplementary Figure 2.

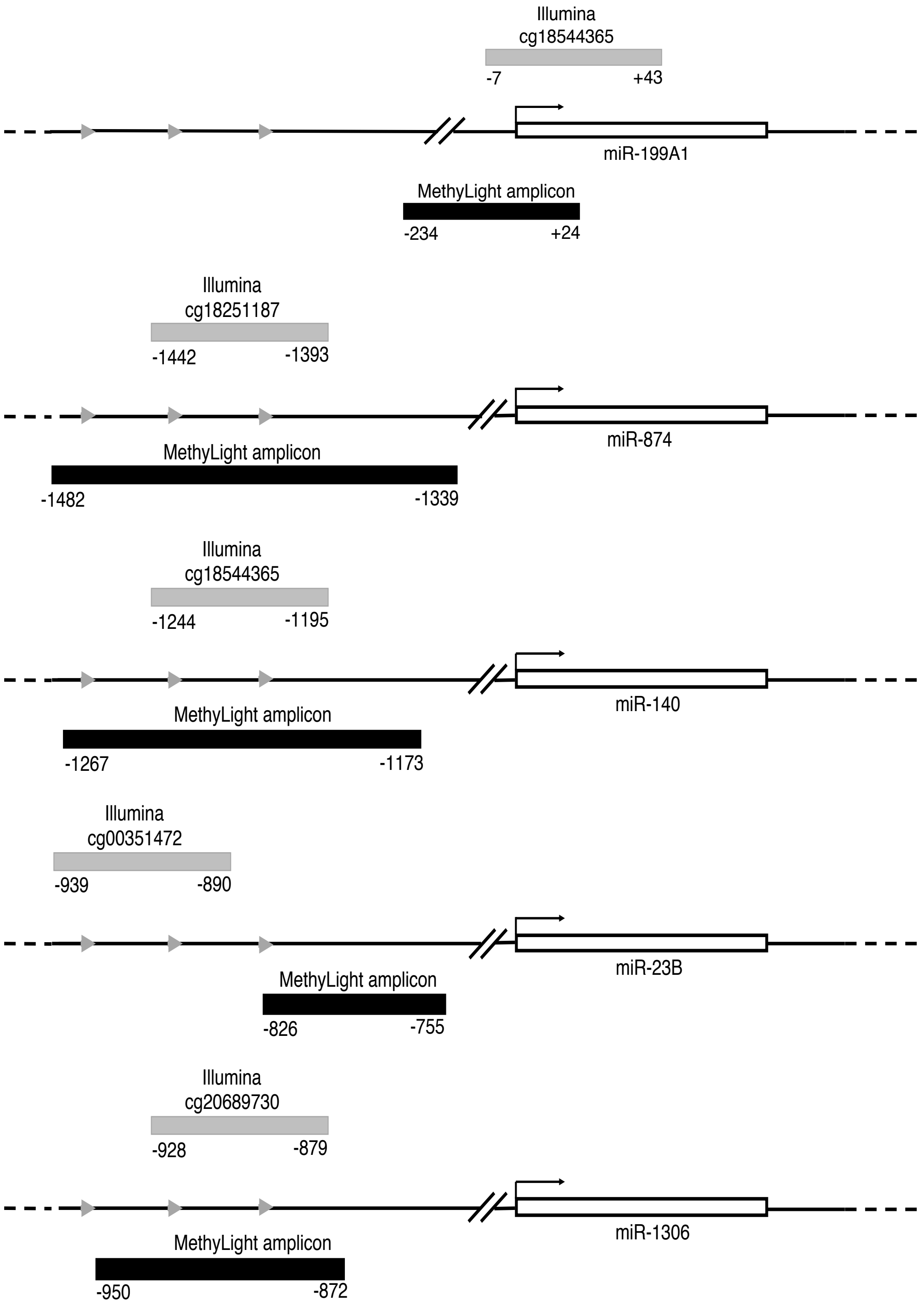

Supplementary Figure 2. The location of Illumina Infinium HM450 array probes used in MethyLight reactions for *miR-199A1*, *miR-874*, *miR-140*, *miR-23B* and *miR-1306* genes.

Supplementary Table 1.

List of MethyLight primer and probe sequences.

Gene	ILMN probes covered	Forward primer sequence	Reverse primer sequence	Probe oligo sequence
miR-199A1	cg18544365	GTAGTTTGAATATTGGGTTGGCG	ACGACCTAACCTAATAACCCCAACG	6FAM-TAAAAAATTCTACAAAATAAATAACCGACC-MGBNF[illegible]
miR-874	cg18251187	GGAATTAGGTTTTTTTTAATATAGTATTCGG	TTAAAATTTAAACCTAAAATAACTAACCCG	6FAM-CTATTTTATTATATTACTTCCAAATATCAC-MGBNF[illegible]
miR-140	cg07281938	AAATTTTATGATTTGCGTCGTCG	CGAAACACTCAAACTCACCTAACG	6FAM-CAAACCCTCCTCGCCACGCATAATAAT-BHQ-1
miR-23B	cg00351472	AAAGGGAAACGGGATTGAGC	CGTCCTAAAAAAAAACCGCG	6FAM-CTACAACAAACGTTTCGCTACG-MGBNFQ
miR-1306	cg20689730	TAGTACGTTGTATTATTGTTTTCGTTTACG	ACGACCTAACCTAATAACCCCAACG	TACTACCCAAAAACCCCAAAA-MGBNFQ

Supplementary Table 2.

Demographics of study participants for MethyLight analysis.

	All FECD (n = 12)	Control (n = 9)	Analyzed FECD (n = 10)
**Age (SD)**	72.12 (8.19)	56.11 (9.05)	73.07 (6.36)
Age range	54.7-85.1	36-67	66.1-85.1
**Sex**			
Male	7 (58%)	2 (22%)	6 (60%)
Female	5 (42%)	7 (78%)	4 (40%)
**Study eye**			
Right	3 (25%)	6 (67%)	2 (29%)
Left	9 (75%)	3 (33%)	8 (71%)
**Procedure**			
DSAEK	2 (17%)	N/A	1 (10%)
DMEK	10 (83%)	N/A	9 (90%)
**FECD measures**			
Guttae (SD)	2.7 (0.78)	0	2.8 (0.82)
Pachymetry (SD)	632 (126)	N/A	626 (135)
